# The Incisive Canal: A Comprehensive Review

**DOI:** 10.7759/cureus.3069

**Published:** 2018-07-30

**Authors:** Sasha Lake, Joe Iwanaga, Shogo Kikuta, Rod J Oskouian, Marios Loukas, R. Shane Tubbs

**Affiliations:** 1 Anatomical Studies, St. George's, St. George, GRD; 2 Medical Education and Simulation, Seattle Science Foundation, Seattle, WA, USA; 3 Seattle Science Foundation, Seattle, USA; 4 Neurosurgery, Swedish Neuroscience Institute, Seattle, USA; 5 Anatomical Sciences, St. George's University, St. George's, GRD; 6 Neurosurgery, Seattle Science Foundation, Seattle, WA, USA

**Keywords:** incisive canal, nasopalatine canal, maxillary incisive canal, dental implant, nasopalatine nerve block, incisive canal cysts

## Abstract

The incisive canal, also known as the nasopalatine canal, is an interosseous conduit through the anterior maxilla connecting the oral and nasal cavities. Within this canal lies the nasopalatine nerve and the vascular anastomosis between the greater palatine and sphenopalatine arteries. The embryology of the canal has led to interesting theories explaining its function. Efforts have been made to describe the morphometrics of the incisive canal by radiologic evaluation across sex and ethnicities. This paper aims to review the current literature on the embryology, anatomy, and clinical relevance of the incisive canal.

## Introduction and background

The incisive canal is located in the anterior part of the hard palate and serves as a communication between the oral and nasal cavities. Soft tissue and neurovascular structures, namely, the nasopalatine nerve and sphenopalatine artery, traverse the length of this canal. Cone-beam computed tomography (CBCT) studies have revolutionized craniofacial imaging and aided in understanding the anatomical and morphometric variability of the incisive canal. Studies of the incisive canal using CBCT have shown, for example, that age, sex, ethnicity, tooth loss, and trauma alter the metrics of the incisive canal. These unique challenges have led to special considerations and novel surgical techniques in oral–maxillary surgery and dental restoration [1-3].

## Review

Embryology and development of the incisive canal

From the fourth to 10th weeks of life, the external facial features of the embryo begin to form through a series of highly intertwined genes and cellular migration events [4]. During this period, the anterior oral cavity commences to separate from the nasal cavity via palatogenesis with the formation of the primary and secondary palates. By the sixth embryonic week, the primary palate will be formed by the fusion of the medial nasal processes [4]. The definitive structures arising from the primary palate are the nasal septum, the pre-maxillary bone, the central incisors, and the upper lip [4-6].

As the primary palate forms, the maxillary processes, arising from the first pharyngeal pouch, produce vertical mesenchymal tissue outgrowths called palatal shelves. These palatal shelves will eventually orient in a horizontal plane, tenting over the tongue, and continue growing until they meet at the midline at the rostral ends of the developing oral cavity [4]. Collectively, the fusion of these palatal shelves results in the formation of the secondary palate. The complete fusion of the primary and secondary palates occurs at the 12th embryonic week [4-7].

Studies on the development of the incisive canal during embryogenesis, as well as the occasionally associated nasopalatine duct, have yielded controversial results. Traditionally, the incisive canal’s development was theorized to be at the central point of fusion between the primary and secondary palates, where a triangular wedge forms. This theory holds that the incisive canal represents an unusual and a rare form of a cleft palate. There are published case reports on adult human cadavers supporting the theory of the incisive canal being a fruste cleft palate [8]. Furthermore, modifications of the cleft palate classifications also include the involvement of the incisive foramen as an extensive form of the submucosal cleft palates [8-11]. Conversely, using seven human embryos, at weeks seven to 24, Radlanski et al. demonstrated that during embryogenesis, the incisive canal was derived from the primary palate within the pre-maxillary bone [5]. In the study, the development of the neurovascular structures within the incisive canal was traced using histology and 3D reconstruction. Radlanski's results strongly emphasized the accepted concept that the nerves and blood vessels are derived from the mesenchymal tissue. The formation of the nasopalatine arteries and nerves at the triangular wedge would not be possible, as it would mean that these structures freely grew in the oral cavity and not within the mesenchyme [5]. In another small observational study by Falci et al., using five fetuses between weeks eight and nine, the development of the incisive canal within the pre-maxillary bone was also confirmed [6]. Using a larger sample size of 26 human fetuses, Kim et al. verified Radlanski’s argument of the incisive canal developing within the pre-maxillary bone by observing that the nerves and blood vessels grew within the mesenchyme, albeit anterior to where the definitive canal is positioned [7].

Occasionally, another structure called the nasopalatine duct can be found inside of the nasopalatine canal. Based on embryology and phylogenetics, the nasopalatine duct in humans is thought to be a vestigial structure. In other mammals, however, the nasopalatine duct transduces signals from pheromones [12]. Radlanski et al. discredited the associations of the nasopalatine duct with the incisive canal. The results from their study put forward that the nasopalatine duct developed within the lateral fusion areas of the primary and secondary palates. Contrasting conclusions have been published in the study by Falci et al. regarding the association of the incisive canal and nasopalatine duct. Falci shared that the nasopalatine duct was located within the canal, adding that the duct can remain patent and continuous, occluded, or segmented [6].

Gross anatomy of the incisive canal

**Figure 1:**
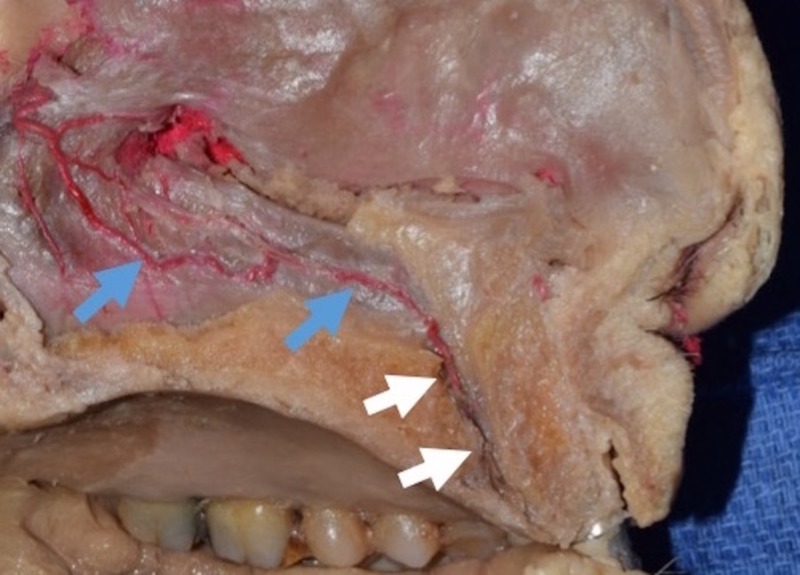
Midsagittal plane of the cadaveric head. Note the posterior septal branch of the sphenopalatine artery (blue arrows) passes through the incisive canal (white arrows).

The incisive canal (also referred to as the nasopalatine canal or anterior palatine canal) is a bony conduit of the maxillary bone connecting the nasal and oral cavities (Figure 1) [5]. Neurovascular structures traversing this canal include the nasopalatine nerve providing sensory input to the pterygopalatine ganglion, from the mucosa of the hard palate and gingiva around the incisors up to the canine teeth and a vascular anastomosis between the posterior septal branch of the sphenopalatine artery and the greater palatine artery [13]. Infrequently, the nasopalatine duct is an additional structure of the incisive canal [12].

The incisive canal terminates at the incisive fossa in the oral cavity, posterior to the maxillary incisors and beneath the incisive papilla. Superiorly, it extends to the nasal cavity on each side of the nasal septum as nasal foramina or Stenson’s foramina, approximately 2 cm posterior to the inner margin of each nostril [12-13]. Given the nature of bilateral superior terminations and the single endpoint at the incisive fossa, the canal has a characteristic “Y” or “V” shape. Fukuda et al. also demonstrated that the “Y” shape was most common, representing 60% of the population [14]. Based on the study by Bornstein et al., there are other alternative shapes classified as types A, B, and C. Type A represents a single canal with no superior duplications in the nasal cavity. The incisive canal also exists as parallel canals that do not coalesce at any point, thus defining the type B formation. Lastly, there may be multiple terminal points alongside the nasal septum, defining the type C morphology [15].

Song et al. documented that the number of arteries was proportional to the number of canal openings in the nasal cavity, which were located centrally and along the lateral walls of the incisive canal [16]. The nerve bundles and veins showed no correlation with the canal openings. The veins also traversed the canal centrally and laterally and were numerous, while nerve bundles remained centrally oriented in the incisive canal with more than two bundles present at each opening [16].

Radiologic evaluations of incisive canal anatomy

**Figure 2:**
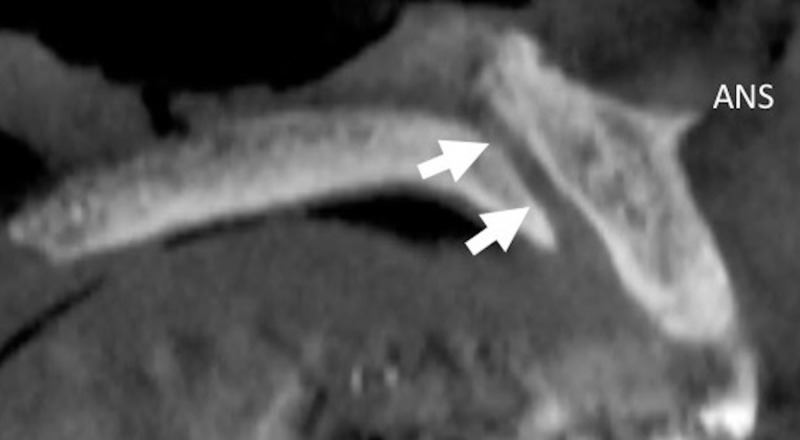
Midsagittal plane of the incisive canal (arrows) on CBCT image. ANS: anterior nasal spine; CBCT: cone-beam computed tomography

**Figure 3:**
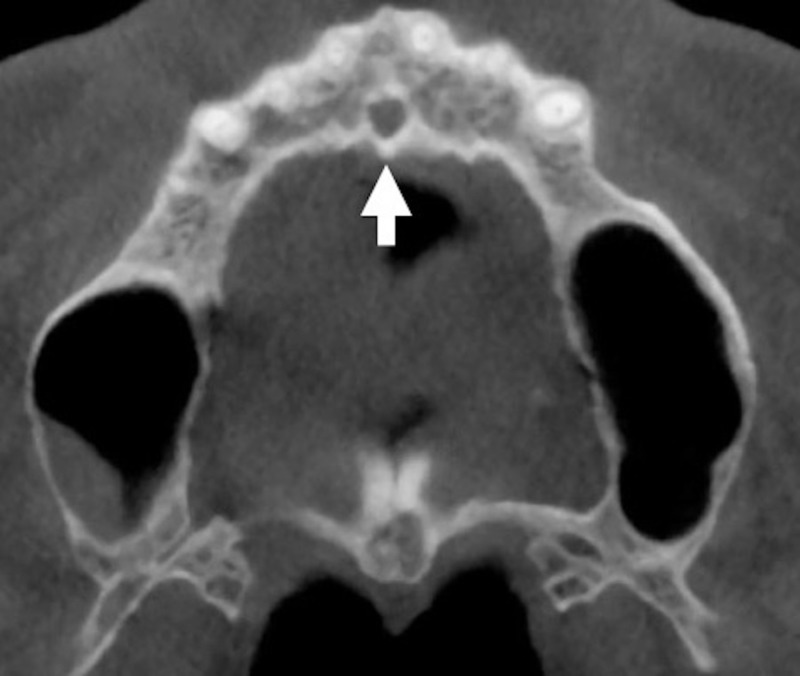
Axial plane of the incisive canal (arrow) on CBCT image. CBCT: cone-beam computed tomography

Craniofacial radiography is essential for the assessment of gross pathology or anatomy interrogation for the planning of craniofacial surgery or restorative dental procedures. Imaging modalities of the maxillofacial region include X-rays with periapical or panoramic views, CT, and magnetic resonance imaging (MRI). Of these, CBCT is the best way to evaluate the incisive canal giving better high-resolution images, eliminating image superimposition, offering less radiation exposure, and providing better analysis of bone quality (Figures 2, 3) [1,2,17].

Radiological imaging methods evaluating the incisive canal further corroborate morphological variances in shape, course, angulation, and direction. On imaging, the shape of the canal has been observed most commonly to be a funnel or Y-shape [14]. Other shapes on radiographic imaging include cylindrical, banana, spindle, or hourglass. The prevalence of these configurations is suggested to vary across ethnicities; based on a study of 63 individuals from a Lebanese population, the cylindrical and funnel forms are prevalent [18]. However, among 301 Iranians, the funnel shape was most commonly observed [19]. On radiographs, the incisive fossa appears as a central radiolucency between the roots of the central incisors. This radiolucency may be: round, oval, lobular, or heart-shaped, depending on the superimposition of the anterior nasal spine [20].

The morphometry of the canal considers the length, diameters at the nasal and oral openings—i.e., at the nasal foramina and incisive fossa, respectively—and thickness of the buccal bone plate over the nasal and oral openings [20]. The length of the incisive canal is measured as the maximum vertical distance between the nasal and oral openings and is an average of 10 mm. Across the literature, evidence both supports and refutes sex differences in the length of the incisive canal. Khojastepour et al. showed statistically significant differences in the length of the incisive canal between males and females where the mean lengths of the canals were 11.46 mm and 9.37 mm, respectively. Although limited by a small sample, the previously mentioned Lebanese study found that the length of the canal was shorter in females [3,21-23]. Additionally, the length of the canal also varies based on the height of the maxillary bone [22].

The diameter of the incisive foramen is on average less than 6 mm in the dentulous maxilla. This measurement is consistent across ethnicities while some also report minor differences between the sexes. Khohastepur et al. reported that the diameter of the nasal and oral openings was higher in men compared to women [20]. It also appears that the diameter increases in pathologies that result in structural deficits of the maxilla such as trauma, cyst formation, and tooth loss.

The buccal bone plate thickness anterior to the incisive fossa from a clinical standpoint is of interest. CBCT studies have demonstrated that maxillary tooth loss markedly reduces buccal bone plate thickness. Interestingly, this finding has been used to explain, with statistical significance, why the incisive canal’s width is positively correlated and its length negatively correlated with tooth loss/extraction and the aging of dentulous maxillae [3,13,15,20,24]. Khohastepur et al. also pinpointed gender variances reporting that the alveolar bone thickness is higher in men than in women [20]. However, this study further mentions that the thickness of the buccal bone plate anterior to Stenson’s foramina was of greater thickness in women compared to men [20].

Morphological depictions of the direction and course of the canal from sagittal imaging views are described as vertical, vertically curved, slanted straight, or slanted curved [22]. Regarding the angulation of the canal, Song et al. reported the slanting angle of the canal, i.e., the angle taken from a vertical reference point, ranged between -7.4 and 35.3 degrees [16]. Here, a negative angulation implies that the incisive foramen terminates more posteriorly than the nasal foramina (Stenson’s foramina) [16].

Clinical aspects of the incisive canal

Dental Implants

Maxillary incisors hold high aesthetic merits and relevance for daily speech function and food consumption. Trauma, especially with palatal bone displacements, periodontal disease, dental extractions, cysts, and tumors, can inevitably lead to tooth loss and impair the bony architecture of the maxilla. The maxilla is more trabecular compared to the mandible and more prone to progressive reabsorption [24-25]; consequently, this alters the morphometrics of the incisive canal [14,26]. This process can result in potential osseointegration failure of the endosseous dental implants and can progress to aesthetically compromising implant angulations from stress biomechanics [27]. Unfortunately, the nasopalatine nerve and accompanying artery are also predisposed to injury during restorative dental procedures. There is the risk of sensory loss to the anterior palate due to damage of the nasopalatine nerve; although temporary, it can go unnoticed as the greater palatine nerve provides an overlapping innervation.

Older reports opted for a complete removal of the nasopalatine nerve and artery within the incisive canal and the placement of dental implants through the canal. Moreover, as an alternative to bone grafting in patients with severe maxillary alveolar ridge atrophy, implants can be anchored around the remaining bone of the incisive canal for additional support. The current literature extensively reports on the problematic nature of the incisive canal and dental implants. Various studies have shown how reducing the width of the incisive canal with various bone grafting materials has proven to provide additional boney support for dental implants and also preserve the integrity of nasopalatine arteries and nerves [25-28]. Artzi et al. described a method where bone graft was used to partially obliterate the diameter of the incisive canal [26]. In this particular case, the incisive canal and fossa were projected to overlap with the proposed implant osteotomy site. Artzi et al. obliterated the canal with bone graft material, and they were able to avoid detrimental manipulations to the canal, which would have injured the nasopalatine nerve and the related artery. The neurovascular bundle was therefore positioned posteriorly within the canal, and the patient suffered no sensory deficits. This maneuver provided an additional space for the osteotomy. Moreover, the dental implant achieved full osseointegration without complications. A case series of five patients by Raghoebar et al. reported a 100% success rate with augmenting the palatine structural bone deficits at the incisive canal with bone grafts. A few patients in this case series reported sensory palatal changes that were subsequently resolved [25]. Veradi and Pastaga also reported two successful cases of canal obliteration with bone ridge augmentation without complications [28].

Nerve Block

The nasopalatine nerves provide sensory innervation to the nasal septum and anterior mucosa of the hard palate and palatal gingiva up to the six anterior teeth, where it overlaps with the branches of the greater palatine nerves. The maxillary incisors are primarily innervated by the anterior superior alveolar branches of the maxillary nerves. Blockade to the anterior superior alveolar branches may not always be successful given the variations in the nasopalatine terminal branches innervating the maxillary central incisors. Nonetheless, the blockade of the nasopalatine nerve is useful for incisor teeth extractions [29].

A landmark for the nasopalatine nerve block is the incisive papilla at the posterior borders of the maxillary central incisors. The needle is inserted at a 45º angle just lateral to the incisive papilla with the bevel toward the palatal mucosa [29]. Nasopalatine nerve blocks are painful due to the nature of the mucosa overlying the incisive fossa. Some suggest using a 30-gauge needle with minimal dead space to ameliorate discomfort [29].

Epistaxis

Nosebleeds occur frequently and have a 60% prevalence in the general population [30]. Eighty percent of nosebleeds are anterior and involve the Little’s area—the anastomosis between the posterior septal branch of the sphenopalatine artery, the nasal septal branch of the superior labial artery, and the branches of the anterior ethmoidal artery. Bleeding is usually managed conservatively; however, intractable bleeding warrants surgical intervention by cauterization, ligation, or embolization. Surgical interventions usually address posterior septal branches of the sphenopalatine artery or the maxillary artery (IMAX ligation: Caldwell–Luc approach) [30].

Butrymowicz et al. described a unique approach to epistaxis: endoscopically cauterizing the sphenopalatine artery via an endonasal approach at the incisive foramen [30]. Complications include septal perforation, septal hematomas, and dental and palatal anesthesia. One of the patients in their study suffered a small septal perforation that was attributed to the preexisting friable mucosa. All patients in this study also remained free of nose bleeds for 24 months after the procedure [30]. The procedure was performed under general anesthesia, and the nasal septum was infiltrated with 5 mL of 1% lidocaine. Each nare was then packed with oxymetazoline pledgets and then removed once adequate decongestion was achieved. Next, a zero-degree rigid nasal endoscope was inserted, and a vertical hemitransfixion incision was made near the caudal end of the cartilaginous septum. The incision was carried down to the floor of the nasal cavity, and a subperichondral flap was raised carefully to preserve the septal mucosa. The flap was raised posteriorly and inferiorly until the region of the incisive foramen was visualized. Elevation of the mucosa continued beyond the foramen for complete visualization and cautery was applied. The septal flap was then laid back in place and sutured using interrupted 4.0 chromic sutures. The nasal splints were trimmed and fitted for the anterior septum and then sutured in place using a mattress 2.0 prolene suture [30]. The authors also suggested that the anterior nasal spine be used as a reliable landmark for the depth of the incisive canal, as it was consistently located within 1 cm from the anterior nasal spine on cadaveric specimens and radiographs [30].

Nasopalatine Cyst

The diameter of the incisive canal measures up to 6 mm; if the diameter exceeds this value, then a canal cyst should be suspected [31-32]. First described by Meyer in 1914, an incisive (nasopalatine) canal cyst is the most common non-odontogenic cyst of the oral cavity accounting for 10% of gnathic bone cysts and occurring in one in 100 persons [32-34]. These cysts carry a slight male predilection and present in the fourth through sixth decades of life [31,33]. However, age should not be used to exclude incisive canal cysts in the younger population, as there have been case reports of nasopalatine cysts in children as young as seven years old [35]. There is no consensus on the etiology of the incisive canal cyst. Some propose that the nasopalatine cysts develop from the spontaneous proliferation of the remnants of embryonic tissue (i.e., the nasopalatine duct) [32]. Previous trauma, poorly fitting dentures, local infection, genetics, and ethnicity are other factors proposed to explain the development of nasopalatine canal cysts [32-33]. The placement of dental implants is reported to aggravate the rapid growth of asymptomatic cysts, which can compromise the integrity of dental implants within proximity [31].

Clinically, most cysts are asymptomatic with the clinical presentation of a fluctuant rounded swelling of the midline anterior hard palate [32]. If symptoms of pain, drainage, pruritus, fistula, or ulceration are present, they usually represent an underlying infection of a previously asymptomatic cyst [31]. A large cyst can distort teeth architecture, destroy the surrounding bone, and invade the nasal cavity floor [31]. On radiographs, cysts appear well-circumscribed anterior to maxillary radiolucencies that are heart shaped, due to the superimposition of the anterior nasal spine. Cysts can also appear as round- or oval-shaped on radiographs [34].

Histopathological analyses reveal a cyst wall lining with a stratified squamous epithelium. However, a combination of stratified squamous epithelium with pseudostratified columnar epithelium with or without accessory cilia or goblet cells, simple columnar epithelium, and simple cuboidal epithelium can be present. The wall can also contain fibrous tissue, veins and nerves, minor salivary glands, and cartilage [35].

Incisive canal cysts are treated with complete surgical removal by a palatal approach with the palatal flap [33]. Before removal, radiographic films with periapical, horizontal angulations, and panoramic and occlusal views should be ordered to assess the nature of the lesion adequately. CBCT also assesses this lesion with better precision and limits radiation exposure [2,34]. Pulp vitality of the surrounding teeth must be thoroughly assessed to further rule out cysts of odontogenic etiology, for example, incisive root cysts [31]. Surgical enucleation of the cyst is performed under local anesthesia and, usually, the cyst is aspirated before removal [31-32]. The differential diagnosis for incisive canal cyst includes: medial enlarged nasopalatine duct, central giant cell granuloma, central incisor root cyst, supernumerary tooth follicular cyst (normally mesiodens), primordial cyst, nasoalveolar cyst, osteitis with palatal fistulization, and bucconasal and/or buccosinusal communication, and intraosseous schwannoma [33,34,36]. It is recommended that cysts be followed up with radiographic images [31,33].

Supernumerary Mesiodens

A mesiodens is a supernumerary tooth that is present at the midline between the two central incisors. Most often, mesiodens are located palatally or within the alveolar process [37-38]. Mesiodens are less frequently associated with the cortical bone of the nasal floor or incisive canal. Mossaz et al. observed that 20.5% of mesiodens are in contact with the cortical bone of the nasal floor, while 49% are in relation with the incisive canal and fall under three categories: i) 38.8% being in external contact with the canal; ii) 8.2% perforated the canal; and iii) 2% located within the canal [37]. Without the symptoms or complications of tooth impaction, tooth crowding, ectopic eruption, root resorption, and cystic lesion formation, mesiodens of the incisive canal can go unnoticed and are incidental findings on radiographs [38].

Cleft Palate

Embryology of the incisive canal places its origin within the primary palate. The initial classification system (Kernahan “Y” classification) used the incisive canal as a landmark for a cleft palate corresponding to the point where the three lines of the "Y" join, and the numbers along the lines denote the zone in which the cleft is located [9]. This system failed to include classifications of the submucosal cleft palate, which can involve the incisive canal. In 1998, the Kernahan “Y” classifications were modified by Smith et al., featuring more details that included descriptions of the cleft region, the site of the cleft, the degree of the cleft, and rare and asymmetrical clefts. In this system, the submucosal cleft palate is denoted by the number "7" and has four alphabetic subdivisions: a, b, c, and d. Type 7 (a) represents the involvement of the primary hard palate lying anterior to the incisive foramen and posterior to the alveolus, (b) the involvement of the palatine process of the maxilla of the secondary hard palate, (c) the involvement of the maxillary process of the palatine bone of the secondary hard palate, and (d) submucosal cleft palate including an occult submucosal cleft palate [11]. The incisive canal should not be regarded as a variation of cleft palate; however, it carries special considerations when determining the degree of palatal dysmorphogenesis [9-11].

Diversity in the anatomy of the incisive canal requires consideration and planning of surgical procedures. Therefore, the variations in this region are essential for the oral surgeon [39-41].

## Conclusions

On average, the incisive canal has a length of 10 mm and a width of up to 6 mm at the incisive fossa, takes the “Y”-shaped morphology, and is located at about 2 cm from the opening of the nares. Morphometric differences are also proposed to vary based on sex and ethnicity. Consistently, the dimensions of the canal are shown to change with the loss of dentition, age, and trauma. Analysis of the width of the canal at the incisive fossa and the structural integrity of the bone ridge anterior to the canal carries high importance to avoid neurovascular damage to the nasopalatine artery and nerve. Achieving the osseointegration of dental implants at the anterior maxilla is highly dependent on the anatomy of the incisive canal. It is also vital to maintain aesthetics, phonation, and avoidance of implant misalignment due to stress biomechanics. Nerve blocks of the nasopalatine nerve at the canal can be applied for dental extractions of central incisors with modification of the technique to alleviate discomfort. Endonasal cauterization of the sphenopalatine artery, as it enters the incisive canal also demonstrates an alternative for stopping intractable anterior epistaxis.
